# Coupling groundwater modeling and biological indicators for identifying river/aquifer exchanges

**DOI:** 10.1186/2193-1801-3-68

**Published:** 2014-02-05

**Authors:** Didier Graillot, Frédéric Paran, Gudrun Bornette, Pierre Marmonier, Christophe Piscart, Laurent Cadilhac

**Affiliations:** UMR 5600 Environnement Ville et Société, CNRS, Ecole Nationale Supérieure des Mines de Saint-Etienne, 158 Cours Fauriel, F-42023 Saint-Etienne Cedex 2, France; UMR 5023 Ecologie des Hydrosystèmes Naturels et Anthropisés, CNRS, Université de Lyon, Université Lyon 1, 43 Boulevard du 11 novembre 1918, F-69622 Villeurbanne Cedex, France; UMR 6553 ECOBIO, Campus Beaulieu, CNRS, Université Rennes 1, 263 Avenue du Général Leclerc, F-35042 Rennes Cedex, France; Agence de l’eau Rhône-Méditerranée & Corse, 2-4 allée de Lodz, F-69363 Lyon, France

**Keywords:** Groundwater, Hydrological modeling, Aquatic vegetation, Aquatic invertebrates, Hyporheic zone

## Abstract

Future climate changes and the resulting modifications in anthropogenic activities will alter the interactions between rivers and groundwater. The quantification of these hydraulic interactions is absolutely necessary for achieving sustainable water use and requires accurate analytical methodologies. This report proposes an interdisciplinary approach to the quantitative and qualitative characterization of hydraulic interactions between rivers and shallow aquifers, wherein it outlines the advantages of coupling groundwater modeling with biological markers. As a first step, we built independent diagnostic maps of hydrological exchanges at the sector scale on the basis of hydrogeological modeling and biological indicators. In a second step, these maps were compared to provide a quantitative and qualitative understanding of exchanges between groundwater and surface water. This comparison significantly improved the calibration of groundwater models through a better assessment of boundary zones. Our approach enabled us to identify the conditions under which it could be possible to use biological indicators instead of a large set of piezometric measures. The integration of such combined tools in a future decision support system will assist governmental authorities in proposing appropriate long-term water policies for the preservation of groundwater resources, such as for supplying potable water and/or mitigating pollution risks.

**Electronic supplementary material:**

The online version of this article (doi:10.1186/2193-1801-3-68) contains supplementary material, which is available to authorized users.

## Introduction

The importance of surface water/groundwater exchanges that are related to i) river discharges, ii) the availability of potable water, iii) river thermal regimes, iv) river productivity regarding both nutrient inputs and interstitial fauna biomass and v) the functioning and dynamics of alluvial wetlands has been frequently outlined (Bravard et al. 1997; Hayashi and Resenberry 2002; Lucassen et al. 2005; Jansson et al. 2007). The assessment of groundwater fluxes between aquifers, rivers and their floodplains thus represents a relevant issue for the long-term management of water resources. A precise delineation of the sections of aquifers that are mostly supplied by rivers and of river sections and floodplain areas that are mostly fed by groundwater upwellings (Bornette and Arens 2002) may constitute a valuable tool for watershed managers to identify strategic water reserves (Griebler et al. 2010). Although some methods exist for this type of quantification (GICC Research Group 2005), they have two main drawbacks. First, building a groundwater model based on a very large-scale grid is time consuming. Second, the amount of synchronous data that are necessary to feed and fit the model is prohibitive. In the framework of global change, the preservation of water and the functions that are associated with groundwater/surface water exchanges requires new methodologies or combinations of existing methods. Such methods should lead to more sustainable water usage and, consequently, for the preservation of groundwater in good quality and the associated biodiversity throughout large rivers.

The objective of this study was to characterize the hydraulic transfers between a large river and the lateral shallow aquifers. Traditionally, in studies of groundwater/surface water interactions on the sector scale, hydraulic models have been ubiquitously used to locate areas of interactions with aquifers (e.g., the Murray River, Lamontagne et al. 2002; the Snake River, Hortness and Vidmar Hortness and Vidmar 2004; the Jordan River, Holtzman et al. Holtzman et al. 2005 Urbano et al. 2006).

More recently, biological and ecological indicators have been used, e.g., microorganisms (Steube et al. 2009; Stein et al. 2010) and invertebrates (Schmidt et al. 2007; Korbel and Hose 2011). The findings of these studies suggest that faunal assemblages reflect hydrological exchanges.

The originality of our study lies in an interdisciplinary approach that combines scientific knowledge regarding the hydraulic control of groundwater transfers with two biological indicators: aquatic plants and groundwater fauna, for which the ecological requirements with respect to groundwater connectivity are known. This approach was applied to a large floodplain section to test its efficiency in providing the integrative mapping of surface water/groundwater interactions one a large scale (10 to 100-km).

## Materials and methods

The originality of the proposed method lies in its combination of physical and biological methods (Bornette et al. 2008) which will be successively presented and then integrated.

### Site location and description

**Figure 1:**
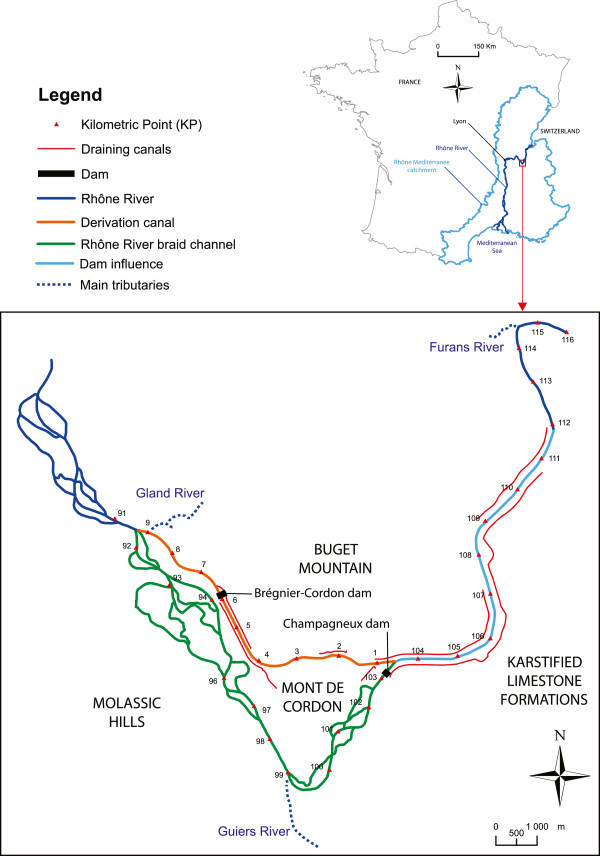
**Location of the Rhône River in France (top) and map of the study site (bottom).** Kilometric points (KP) decrease from upstream to downstream for the main and by-passed channels (from KP 110 to KP 91) but increase in the deviation canal from the dam (KP1) to the main channel (KP 9).

The Rhône River runs from Switzerland in the Alps to the Mediterranean Sea along a 545-km course. The river catchment covers approximately 5220 km^2^ of surface area (Figure 1). Along this river, several types of aquifers occur: free and confined sedimentary aquifers, impervious geological formations and aquifers in fissured bedrock. Many man-made structures have been built along the Rhône River, including approximately 40 dams for hydroelectric power production and flood prevention, navigational enhancement, and the management of water for irrigation (Bravard et al. 1986; Bravard 1987).

Our study focused on the Brégnier-Cordon river stretch (Ain, France). This site was chosen based on three main criteria: i) a low level of human activities; ii) the existence of hydraulic and biological data (Creuzé des Châtelliers 1991; Bornette et al. 1998a) and iii) the existence of previous studies (Michal 1988; Bornette et al. 1994). This river stretch is located 100-km upstream from the city of Lyon from KP116 to KP91 (KP: kilometric distance from a reference point, the Rhône-Saône confluence, thus decreasing from upstream to downstream; Figure 1). The Rhône River flows from the north-east to the south-west, and the slope ranges between 0.7 and 1‰. The average river discharge is approximately 423 m^3^/s, varies from 160 m^3^/s to 1900 m^3^/s, and is governed by a pluvio-nival type regime. The strong slope is associated with plentiful sediment transport from the tributaries of the Pré-Alps Mountains, and a wide range of braided-style flow regimes has developed (Creuzé des Châtelliers 1991; Bravard et al. 1986). The geology of the area is a complex mix of karstic limestone mountains and molassic hills that surrounds a large alluvial floodplain. The left bank of the river consists of partially karstified limestone formations in the eastern part (KP110 to KP100) and a large area molassic hills in the western part from KP99 to the downstream end of the sector. The right bank consists of karstic limestone and marls of the Jurassic period, which is known as Bugey Mountain and stretches from KP105 to the downstream end of the sector. A small karstic Mountain, Mont de Cordon, is isolated between two channels of the river between KP2 and KP3 (Figure 1). The floodplain around the river is a glacial valley that has been refilled with quaternary alluviums. The hydraulic conductivity of these alluviums decreases from 1 × 10–3 to 1 × 10–5 m/s and the thickness close to the Rhône River is approximately 10 m (Chauve 2006).

The river and its floodplain have been considerably modified by a hydroelectric power station that was constructed between 1982 and 1984 (Figure 1). A derivation dam divides the river channel into two branches from KP104 to KP91 (KP103, Champagneux dam). Upstream from this dam, the channel has been transformed into a 12-km long reservoir (KP112 to KP103). Downstream of this dam, the former braided channel (KP103 to KP91) is now a by-passed channel with a regulated discharge that ranges from 80 m^3^/s in the winter to 150 m^3^/s in the summer (Bornette et al. 1994). This by-passed channel is also fed by the Guiers River (KP99). Most of the Rhône River water is diverted by the upstream dam to an 8-km derivation canal (KP1 to KP9) that feeds a hydroelectric facility (KP6) with a maximum fall of approximately 14.3 m (Q = 780 m^3^/s). To collect water that exfiltrates along the banks of the derivation canal and the reservoir, two drainage canals have been dug at the foot of the reservoir and two at the foot of the derivation canal to reduce the potential rise in the floodplain groundwater level (Claret et al. 1999).

### Hydraulic diagnosis

Hydraulic interactions between groundwater and the river were preliminarily estimated through gradients that were calculated from digitalized piezometric maps with GIS spatial analysis tools (ArcGIS 9.2, ArcInfo, ESRI, http://www.esri.com/). The method was founded on the properties of the triangulated irregular network (TIN) model, which is very useful for generating surfaces. Each triangle contains three values as defined in ArcInfo: elevation, slope (or gradient) and aspect (or angle) (Bornette et al. 2008; Paran et al. 2008).

The proposed method permits the characterization of exchanges between any aquifer and river along a linear interface (bank) that can be represented as a polyline consisting of several segments. The exchange characterization implies: i) the quantification of the rate of flow (Q in m^3^/j) and ii) the identification of the direction of hydraulic interactions, wherein fluxes from the aquifer to the river are characterized by positive values (+Q) and exchanges from the river to the aquifer by negative values (−Q). Infiltration (Q: m^3^/s) through the bank was calculated using Darcy’s law (Equation 1) considering gradients (i), the cross-section of the river (A: m^2^) and the permeability of the aquifer (K: m/s).1

The gradient values (i) at the aquifer/river interface were obtained from digitalized piezometric maps (low water level in February of 1990), which were converted in the TIN. Cross-section (A) values were estimated by subtracting groundwater elevation values from riverbed elevation values. Permeability (K) values were obtained from the literature (Michal 1988; Rampnoux 1992; Hydratec Design Office 2002).

The exchange directions were given by the sign (+ or -) of the sinus of the angle formed by the groundwater flow and river flow. This angle (D) was calculated by subtracting the angle values given by the TIN of the piezometric levels and the TIN of the river levels. The infiltration estimation (Qe, Equation 2) was weighted by the value of the sinus of angle D to take into account the influence of the angle between the groundwater and superficial flows on the exchanges. The groundwater flow intensity was usually too weak when Qe was provided in m^3^/s; hence, Qe was given in m^3^/d:2

Qe was calculated for each segment of the Rhône river bank polyline. The final infiltration estimation (Qf) was given by Equation 3. Qf is the sum of each Qe calculated for a segment of the polyline multiplied by the length (L) of this segment:3

This algorithm facilitated a selection of the scale for which the exchange flows had to be calculated. It was then possible to aggregate values for several 1-km-long unitary segments along a predetermined river Section.

### Interstitial invertebrates

The interstitial fauna was sampled within the sediment using the Bou-Rouch technique (Bou and Rouch 1967; Bou 1974), which involves the use of a perforated metal pipe. This mobile pipe was driven 50 cm deep at each sampling point, where 10 L of a mix of interstitial water and sediment was pumped prior to filtering through a 200-μm-size mesh net. Samples were preserved in the field in a 5% formaldehyde solution, stained with Bengal pink, and sorted in the laboratory. A total of 41 sites were sampled in the main active channel of the river, the drainage canals and the wetlands located in the floodplain. They were sampled from 1987 to 2009 with varying periodicity: 16 stations were sampled in the main river channel (sampled twice in 1988 and 1989; Creuzé des Châtelliers 1991) and three were sampled again in 2008 and 2009 for the present study. A total of 16 stations were investigated in the drainage canals [three stations were sampled during the summer in 1987 and 1995 (Claret et al. 1999), six others during the summer in 1995, 1996 and 1997 (Marmonier et al. 2000), and ten others in 2008 (unpublished data)]. Finally, five stations were sampled in abandoned river channels: two stations were sampled in April, July and November in 1987 and 1995, which were years before and after the restoration of the wetland, respectively [fine sediment was removed by dredging in 1993 (Claret et al. 1999), and three others were sampled in 2007 (unpublished data)].

To locate groundwater upwelling zones, we used the absolute and relative taxonomic richness (S and S%) and the abundances (Q) of the stygobite fauna. We used abundances and richness because the stygobite fauna is more abundant and diverse in upwelling zones than in downwelling zones (Marmonier and Dole 1986; Dole-Olivier and Marmonier 1992). We also considered relative richness values because in areas fed by groundwater inputs, the ratio between stygobite and epigean species is expected to increase in interstitial assemblages and should consequently represent a relevant indicator for hydrological exchanges.

### Aquatic vegetation

Aquatic vegetation is strongly governed by the groundwater/surface water connectivity in aquatic ecosystems (Bornette and Amoros 1991; Bornette and Large 1995; Bornette et al. 1998b). The distribution of aquatic plant species is related to the nutrient levels (Carbiener et al. 1990,1995 ; Onaindia et al. 1996; Britto et al. 2001) and seasonal thermal variation in the water (Bornette and Puijalon 2009,2011). In aquatic habitats, there is a strong correlation between the intensity of groundwater exfiltrations and seasonal temperature variability (Sakura 1993; Bornette and Large 1995). The various water sources that supply aquatic habitats usually strongly contrast in terms of nutrient levels (Bornette et al. 1998a; Bornette and Arens 2002). Knowledge of the ecological requirements of species that occur in a given habitat allows the determination of the origin and turnover rate of the water that supplies the habitat (Amoros et al. 2000). For this purpose we had to i) identify the ecological range of aquatic species in terms of trophic levels in the water and temperature, ii) map the occurrence of aquatic species in the aquatic habitats of the floodplain and iii) determine the weight of groundwater supplies in each of these aquatic habitats and their potential origin, as estimated using the abundance and ecological requirements of the aquatic species.

#### Identification of the ecological range of aquatic species

The ecological range of each species was based on the occurrence of a significant relationship between species occurrence and relative abundance and i) the thermal regime and ii) the water trophic levels of wetlands. For this purpose, we used floristic relevés that were collected from 1993 and 1999 in 36 wetlands of the Rhône and Ain Rivers. The physico-chemical characteristics of the wetlands were simultaneously measured monthly for at least a year in the same set of wetlands (i.e., 12 replicates in the Rhône River wetlands and 24 for the Ain wetlands in terms of ammonia nitrogen, phosphates and temperature). This sampling was performed in the same year as (Rhône River) or the same year and year after (Ain River) vegetation sampling. At the same time, three to six substrate samples were collected in all of the wetlands to determine the phosphorous level of the substrate. Ecological profiles were produced for all species occurring in at least five sites among the 36.

Cool, well-oxygenated groundwater supplies limit plant growth and competition processes but also allow evergreen species to grow throughout the year (Bornette and Large 1995; Greulich and Bornette 2003). Consequently, the distribution of aquatic plants is partly ruled by water temperature. To quantify the groundwater supplies to the wetlands, the relative standard deviation of temperatures was calculated for each wetland, and the wetlands were divided into five equilibrated classes according to this coefficient. In a second step, the average abundance of each species for all of the wetlands belonging to a given class was calculated. The difference between the classes was then tested for each species. First, the equality of variances between classes was tested using a Levene test. Second, either parametric or non-parametric tests were performed to determine whether the abundance of species significantly differed between classes.

Combined with knowledge about groundwater supplies, the nutrient content of the water in the wetlands provides information about the origin of groundwater, that is, the seepage water is nutrient rich in the Brégnier-Cordon area, whereas the karstic lateral aquifers should be less eutrophic (Bornette et al. [Bibr CR7][Bibr CR8]). First, the species affinity for phosphorous (both in the water [PO_4_^3-^] and the substrate total P content) was assessed. For this purpose, monthly [PO_4_^3-^] values were log transformed and averaged per site, and the total phosphorous values were standardized and log transformed. Then, both values were summed for each site, and the same method that was used for temperature was applied: wetlands were categorized into five classes according to their total phosphorous contents, and the non-random character of the species distribution along the phosphorous gradient was tested. The same procedure was applied to the ammonia nitrogen contents of water [N-NH_3_]; however, wetlands were separated into four instead of five equilibrated classes. The species affinity for nitrate nitrogen is not assessed because it has been demonstrated to rarely rely to eutrophication. Phosphorous is frequently the major limiting nutrient for aquatic plants (Amoros et al. 2000; Bornette and Puijalon 2009,2011). The affinities of aquatic species for phosphorus and ammonia nitrogen were then qualitatively combined in a trophic profile of four classes by evaluating the relative affinity of the species for eutrophication. The analyses were carried out with Jump (JMP® Statistical Discovery Software, http://www.jmp.com/).

#### The mapping of the aquatic species occurrence

The aquatic species contents and relative abundances were then roughly determined for each aquatic habitat of the Brégnier-Cordon reach. For this purpose, at least one floristic relevé was carried out in each wetland in an area that was considered to be representative of the floristic contents of each wetland. For rivers, canals and riverine wetlands, several relevés were performed to take into account changes in water quality along the upstream/downstream gradients. For each relevé, the relative abundance of each species was estimated using the Braun-Blanquet (1932) index. Then, the trophic levels were estimated by i) multiplying the abundance of each species by its ecological profile and ii) summing the values obtained for all species of the relevé (Amoros et al. 2000). We thus obtained the affinity of the wetland for each trophic class. We considered the class presenting the highest values to indicate the most probable trophic level (Additional file 1). For groundwater inputs, each species was classified according to its affinity for stenothermic water. The difference between the number of species that were intolerant to stenothermic waters and those preferentially occurring in groundwater-fed ecosystems was determined. Then, the wetlands were mapped according to four groundwater influence classes (null: index value = 0; low: index value < 4; intermediate: index values ranging from 5 to 9; high: index value > 9) and four trophic level classes (oligotrophic, mesotrophic, eutrophic and hyper-eutrophic). A comparison of the two maps then facilitated the identification of several geographic areas with contrasting groundwater influences.

### Map crossed analysis

The three types of describers resulted in the production of three separate sets of maps that all located the shallow aquifer recharge areas by the river and sections of the river fed by groundwater. These maps cannot be directly compared because of their characteristics; however, it was possible to analyze the global patterns that resulted from the three different describers using overlay mapping.

A this stage of our work, we can only analyze the global patterns because the available data were not collected simultaneously. However, the crossed analysis is possible because data were sampled during comparable hydrologic conditions (low water level), during the same decade (February 1990 for piezometric values; summers 1987, 1995, 1996, 1997 and 2008 for interstitial invertebrates data; summers 1993 to 1999 for aquatic vegetation and chemical data) and after the modifications inferred by the hydroelectric power station. Furthermore, we will look if significant correlations can be found between estimated infiltration, stygobite richness and abundance and macrophyte index.

## Results

The diagnoses of the surface water/groundwater exchanges that were made using the three describers were first independently considered for the three types of metrics, and the subsequent three maps were then superimposed.

### Hydraulic modeling

**Figure 2:**
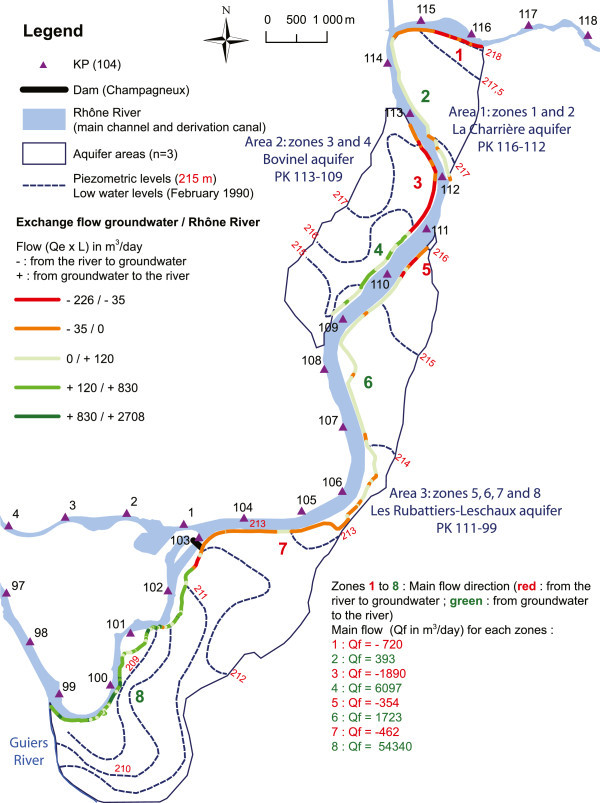
**Mapping of the piezometric levels in the alluvial aquifer and the hydraulic exchanges between the river and the alluvial aquifer.**

Gradients (i) were obtained from the piezometric map TIN (low water level in February of 1990). The average cross-section of the river (A) was approximately 4.2 ± 1.2 m (min = 1.8, max = 8.1). The following permeability values (K) were obtained: Area 1, 1.3 × 10–3 m/s; Area 2, 5 × 10–3 m/s; Area 3, KP111 to KP106, K = 5 × 10–3 m/s, KP106 to KP102.5, K = 3.9 × 10–4 m/s and KP102.5 to KP99, K = 8.5 × 10–3 m/s (Figure 2). The infiltration estimation (Qe) was calculated for each area. For each of the zones with the same global exchange behavior, we calculated the Qf and the unit flow (Qu) for 1-km (m^3^/day/km).

From a hydrogeological point of view, the GIS spatial analysis method was tested on three distinct aquifers to calculate groundwater/river exchanges (Figure 2 and Table 1):

- First, the polyline of Area 1 (the La Charrière zone, approximately 5600 m long, KP116 to KP112) consisted of 62 segments. The results provide evidence that the left bank of the Rhône River can be divided into two zones (Figure 2): zone 1 (KP116 to KP114.5), which is associated with the river supplying the aquifer and a Qf of −668 m^3^/day, and zone 2 (KP114.5 to KP112), which consists of an aquifer drained by the Rhône River and a Qf of 393 m^3^/day.
- Second, the polyline of Area 2 (the Bovinel zone, approximately 4850 m long, KP113 to KP109) was comprised of 74 segments. The results provide evidence that the left bank of the Rhône River to can divided into two zones (Figure 2): zone 3 (KP113 to KP111), with the river supplying the aquifer and a Qf of −1890 m^3^/day, and zone 4 (KP111 to KP109), with an aquifer drained by the river and a Qf of 6097 m^3^/day.
- Third, the polyline of Area 3 (the Les Rubattiers-Leschaux zone, approximately 12400 m, KP111 to KP99) consisted of 312 segments. These results provide evidence that the left bank of the Rhône River can be divided into four zones (Figure 2): zone 5 (KP111 to KP110), with the river supplying the aquifer and a Qf of −354 m^3^/day; zone 6 (KP110 to KP106), with the river draining the aquifer and a Qf of 1723 m^3^/day; zone 7 (KP106 to KP102.5), with the river supplying the aquifer and a Qf of −462 m^3^/day; and, finally, zone 8 (KP102.5 to KP99), with the river draining the aquifer and a Qf of 54340 m^3^/day.

**Table 1:** **Synthesis of the exchange flow between groundwater and Rhône River in the Brégnier-Cordon sector**

Name	Stretch / KP	Flow direction	Qf	Bank length m	Qu
			m^3^/day		m^3^/j/km
**Area 1 (A1)**	Zone 1 (Z1)	-	-668	1547	-432
	116 to114.5				
**La Charrière**	114.5 to 112	-	-52	531	-98
	Zone 2 (Z2)	+	393	3545	111
*Total A1 (Z1 + Z2)*					
*-*			*-720*	*2079*	*-347*
*+*			*393*	*3545*	*111*
**Area 2 (A2)**	Zone 3 (Z3)	-	-1890	2040	-927
	113 to 111				
**Bovinel**	Zone 4 (Z4)	+	6097	2809	2170
	111 to 109				
**Area 3 (A3)**	Zone 5 (Z5)	-	-354	587	-603
**Les Rubattiers-Leschaux**	110.5 to 110	+	5	76	64
	Zone 6 (Z6)	-	-77	431	-178
	110 to 106	+	1723	3709	465
	Zone 7 (Z7)	-	-462	3057	-151
	106 to 102.5	+	8	349	24
	Zone 8 (Z8)	-	-35	21	-1693
	102.5 to 99	+	54340	4190	12968
*Total A3 (Z5 + Z6 + Z7 + Z8)*					
*-*			*-928*	*4096*	*-226*
*+*			*56077*	*8323*	*6737*
**Total A1 + A2 + A3**					
**-**			**-3538**	**8214**	**-431**
**+**			**62566**	**14678**	**4263**

The zones (Z) and stretches (S) are shown in Figure 2.

Overall, the Rhône River in the Brégnier-Cordon sector gains 62566 m^3^/day of water (~4263 m^3^/day/km) and loses 3538 m^3^/day of water (~431 m^3^/day/km). A general finding which is not explicitly mentioned is that the river gained much more water from the aquifer than it lost to the aquifer within the stretch investigated. Indeed, the groundwater discharge into the river was very low (i.e., ~0.72 m^3^/s) in comparison to the Rhône River discharge (i.e., ~160 m^3^/s during the season of low discharge).

### Stygobite fauna

**Figure 3:**
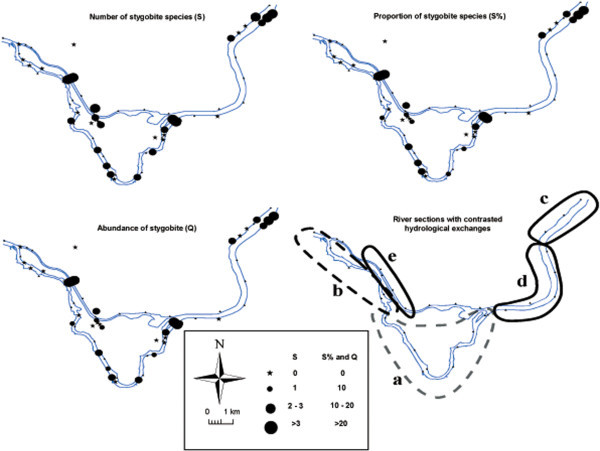
**Maps of the potential exchanges between surface water and groundwater based on the stygobite taxonomic richness (S and S%) and abundances (Q).** The five river sections with contrasting hydrological exchanges are shown for the Rhône River (dashed lines) and for the drainage canals (solid lines).

A total of 106 taxa were sampled, of which nine were stygobite species (Table 2). The local richness, abundance and composition of the stygofauna greatly varied with the channel type and location (Figure 3).

**Table 2:** **List of stygobite species sampled in the Brégnier-Cordon sector between 1988 and 2008**

Order	Species	KP of occurrence
**Mesogastropoda**	*Islamia moquiniana*	3 – 6; 97 – 103
**Ostracoda**	*Fabaeformiscandona wegelini*	97 – 103
**Bathynellacea**	*Parabathynella* cf *stygia.*	96 – 97
**Amphipoda**	*Crangonyx subterraneus*	96 – 97
	*Nipgargopsis casparyi*	4 – 5; 96 – 103; 109 – 110
	*Niphargus fontanus*	4 – 5; 96 – 98; 109 – 110
	*Niphargus kochianus*	4 – 6; 97
	*Niphargus rhenorhodanensis*	96 – 103; 109 – 110
	*Salentinella juberthiae*	96 – 97; 109 – 110

In the by-passed channel, two contrasting sections were distinguished. From the dam to the confluence with the Guiers River and to the beginning of the anastomosed sector (KP103 to KP96), seven stations harbored stygobite species in their interstitial assemblages (Section a, Figure 3). The abundance and richness of stygobite species greatly varied, with locally significant values being obtained, especially for the varied abundance (15 individuals at KP101 and 13 individuals at KP99), suggesting punctual inputs of groundwater into the by-passed channel. Here, the stygobite species consisted of shallow interstitial fauna (*Fabaeformiscandona wegelini, Islamia moquiniana, Niphargopsis casparyi* and *Niphargus rhenorhodanensis*). Downstream of this section (KP91 to KP96), no stygobite fauna was sampled in the by-passed channel, suggesting little or no groundwater inputs there (Section b, Figure 3). This result was consistent with the geomorphological characteristics of this section, which is associated with sediment deposition and clogging.

Additionally, contrasting results were obtained in the four drainage canals (Figure 3). Upstream of the dam (KP112 to KP109), the abundance and richness were high at the upstream end of the right and left side drainage canals, suggesting important groundwater inputs (Section c, Figure 3). Here, the stygobite fauna consisted of two crustacean amphipod species living in shallow interstitial habitats of the main channel (*N. casparyi, N. rhenorhodanensis*) and two species living deeper in the sediment (*Salentilella juberthiae*), or frequently found far from the main channel (*Niphargus fontanus*). This last species may indicate inputs of groundwater from the nearby karstic system on the left bank. From KP109 to KP103, in the area influenced by the reservoir, no stygobites were sampled in the drainage canals, suggesting a weak influence of groundwater in comparison to important inputs of surface water that filtrate through the river banks (Section d, Figure 3). In contrast, in the left and right side drainage canals that are located downstream of the dam (KP4 to KP6), stygobite species were sampled in the entire length of the left canal and at the upstream end of the right one, but with low richness and low abundances (Section e, Figure 3). Here, the stygobite fauna consisted of two amphipod species living in shallow interstitial habitats of the main channel (*N. casparyi, N. rhenorhodanensis*) and four species living deep in the sediment far from the main channel (*N. fontanus, Niphargus kochianus, Crangonyx subterraneus* and *Parabathynella* cf *stygia*), suggesting a mix of surface water infiltrating through the bank and deep groundwater coming from the nearby karstic systems (i.e., Bugey for the right-side canal and Mont Cordon for the left one).

### Aquatic vegetation

The observed aquatic vegetation facilitated the discrimination of four areas. In the first upstream area, the Rhône River flowed as a unique channel along a karstic massif. All of the wetlands were oligotrophic or mesotrophic, with the groundwater influence ranging from low to high. No eutrophic wetlands occurred along the right side of the river, suggesting that the wetlands were not supplied by seepage water but, instead, by a drained hillslope aquifer in this area. Along the left bank of the river, some hyper-eutrophic wetlands occurred along the karstic massif, suggesting either that the wetlands did not drain the hillslope aquifer but, instead, were supplied by river seepage, or that the aquifer was eutrophic in this area. Downstream of this reach, a zone where the wetlands appeared to be eutrophic occurred. Because the river seepage is hyper-eutrophic in this area (Bornette et al. 1994), this pattern could indicate either the drainage of a eutrophic aquifer by the wetlands, or that the wetlands are supplied by a mixture of both oligotrophic groundwater and hyper-eutrophic seepage. This second interpretation was reinforced by the occurrence of oligotrophic wetlands along the floodplain margins. A third zone was discriminated by the braided zone of the river, wherein the sampled vegetation indicated that most wetlands were poorly supplied by groundwater and were hyper-eutrophic, indicating that the river was aggrading in comparison to its floodplain, and that only a few seepage-supplied wetlands occurred. Along the river margin, the wetlands were highly supplied by groundwater and less eutrophic, suggesting some influence of a hillslope aquifer.

### Superimposition of the maps

**Figure 4:**
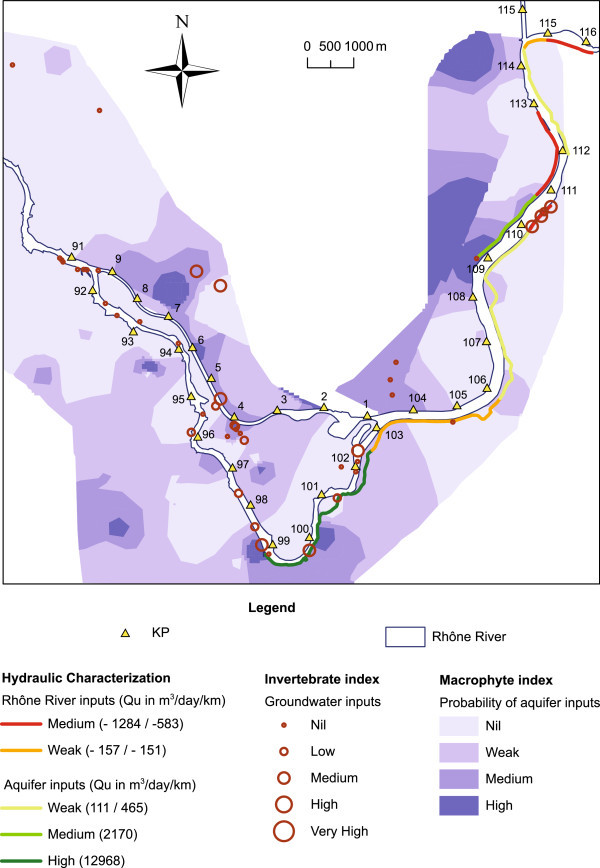
**Comparison map that combines the results from the hydraulic, invertebrate and macrophyte indicators.**

The superimposition of the three maps (Figure 4 and Table 3) highlighted the convergences and divergences among the describers:

- In most cases, the results of the three methods were consistent. In area 1, at La Charrière (left bank, KP114.5 to KP112), there was a lack of groundwater invertebrates; however, hydraulic modeling and the macrophyte index both indicated poor to low (Qu = 111 m^3^/day/km) inputs of groundwater to the river. From KP110 to KP106 (left bank), the macrophyte index and the hydraulic method converged and indicated low intensity drainage of the aquifer by the river (Qu = 465 m^3^/day/km). From KP106 to KP102.5 (on the left bank), the three methods provided similar results, i.e., a low supply of river water to the aquifer (Qu = −151 m^3^/day/km). Finally, the results were also consistent for the KP102.5 to KP99 section (left bank) and indicated medium to high intensity drainage of the aquifer by the river (Qu = 12968 m^3^/day/km). For the right bank, all of the methods provided similar results for the reach located from KP110.75 to KP109, i.e., medium to high intensity drainage of the aquifer by the river (Qu = 2170 m^3^/day/km).
- Discrepancies only occurred between the different methods in two cases. First, in zone 2, at Bovinel (right bank, KP113 to KP111), the macrophyte index did not detect a flow from the river to the aquifer, whereas the hydraulic method did (Qu = −927 m^3^/day/km). Second, in zone 3, at Les Rubattiers-Leschaux (left bank, KP111 to KP110), the three methods diverged over a small distance: the macrophyte index did not highlight groundwater inputs, whereas the hydraulic method indicated a weak flow from the river to the aquifer (Qu = −603 m^3^/day/km) and the invertebrate index indicated an important mixing of surface water and groundwater in the drainage canal.
- Finally, in the zones where no hydraulic diagnosis was available (downstream of KP99 and along the derivation canal), the macrophyte and groundwater fauna indices gave rather similar results. From KP4 to KP9 (right bank, derivation canal) and around Mont de Cordon (left bank, derivation canal), both biological indices indicated groundwater upwelling to the drainage canals and to the river. Downstream of KP99 (left bank) and KP9 (right bank) to the end of the study area, both macrophyte and invertebrate indices indicated a lack of groundwater influences.

**Table 3:** **Comparison of the results of the hydraulic and biological methods**

			Hydraulic		Macrophytes	Invertebrates
KP	Bank	Type	Qf	Qu		
			m^3^/day	m^3^/day/km		
115_114.5	Right	Rhône River	-	-	Weak, karst	-
114.5_113	Right	Rhône River	-	-	Nil	-
113_111	Right	Rhône River	-1541	-871	Nil	-
111_110.75	Right	Dam influence	-349	-1294	Medium to high, karst	-
110.75_109	Right	Dam influence	6097	2170	Medium to high, karst	-
109_108	Right	Dam influence	-	-	Medium to high, karst	-
108_105	Right	Dam influence	-	-	Nil to weak, karst	-
105_103	Right	Dam influence	-	-	Weak to medium, karst	-
1_4	Right	Derivation canal	-	-	-	-
4_9	Right	Derivation canal Restitution	-	-	Medium to high, aquifer of the Gland River	Deep groundwater flow
9_dowstream	Right	Restitution, Rhône River	-	-	Weak to nil	Weak groundwater flow
116_115	Left	Rhône River	-582	-583	-	-
115_114.5	Left	Rhône River	-86	-157	Nil	-
114.5_112	Left	Rhône River	393	111	Nil	-
112_111	Left	Rhône River	-	-	Nil	-
111_110	Left	Dam influence	-354	-603	Nil, pollution from karst	Important exchanges, mixing
110_106	Left	Dam influence	1723	465	Nil to weak	-
106_102.5	Left	Dam influence	-462	-151	Nil to weak, flow from the Rhône River to the aquifer	Flow from the Rhône River to the aquifer
102.5_99	Left	Rhône River	54340	12968	Medium to high, groundwater	Flow from the aquifer to the Rhône River
99_dowstream	Left	Rhône River	-	-	Weak, preferential corridor, groundwater	preferential corridor, groundwater
103_99 / 1_5	Mont Cordon	Rhône River, Derivation canal	-	-	Karstic reappearance	Karstic reappearance

Significant correlations were observed between the three methods (i.e., estimated infiltration (Qu), stygobite richness and abundance and macrophyte index, n = 11 zones). Thus, we found a significant correlation either between the macrophyte index and invertebrate richness (R^2^ = 0.407, p = 0.034) and invertebrate abundance (R^2^ = 0.382, p = 0.042) or between the macrophyte index and the infiltration estimation (R^2^ = 0.503, p = 0.014). No significant correlation was found between the hydraulic infiltration estimation and the groundwater invertebrates.

## Discussion

### Validation elements for hydraulic exchanges

Hydraulic studies have been previously conducted to estimate exchanges between the Rhône River and groundwater in the same area of Brégnier-Cordon based on hydraulic modeling and different piezometric measurements (Rampnoux 1992; Michal 1988; Košmelj 1982) but without the quantification of fluxes. These studies provided hydraulic gradient values for five zones along the right and left banks of the river. The directions of these exchanges were confirmed by the results of the present study. The quantifications of the fluxes that were performed in the present analyses highlighted very weak values that could be associated with groundwater fluxes. This suggests that the Brégnier-Cordon aquifer represents a limited reserve of groundwater and that its use for irrigation or as a source of potable water must be restricted. The multidisciplinary strategy employed in this study could lead to a new modeling approach that can be applied on a broad scale in instances where it would be too costly and time consuming to implement methods that use gridded datasets.

### Multi-metric comparison

#### Convergence of the results

Despite the absence of significant correlation between hydrology and groundwater fauna, we observed a surprisingly good convergence of the results that were obtained using hydraulic and biological indicators. This was especially observable on the left bank of the river from KP106 to KP102.5. In this section, which was influenced by the dam, the estimated flow value was 460 m^3^/day from the river to the aquifer. The value of the macrophyte index was very low and indicated a lack of water exchange between groundwater and surface water. The invertebrate index confirmed the direction of the flow and its intensity in comparison to other sections of the river. Between the dam and the confluence of the Guiers River at KP99, the flow rate value significantly increased until reaching 45.367 m^3^/day from the aquifer to the river. This result was confirmed by both the macrophyte and invertebrate indices.

#### Divergence needing complementary approach

Interestingly, we did not find this convergence at all of the sites. For example, between KP111 and KP110, the hydraulic gradient and macrophyte index demonstrated that the river fed the aquifer; however, the invertebrate indicators highlighted important water exchanges. In this case, further investigations through geochemical and isotopic analyses may facilitate the identification of the origin of the water. The use of environmental isotopic indicators would be a valuable method for validating the results of the present study (Fette et al. 2005). For example, the ratios of isotopic hydrogen (δD) or oxygen (δ^18^O) are usually used for identifying river/aquifer exchanges (Gonfiantini et al. 1995; Walker and Krabbenhoft 1995). This approach has been used on other sections of the Rhône River to identify the origin of surface-water and groundwater (Paran et al. 2012).

#### Conclusion about multi-metric comparison

Moreover, a combination of these three methods provides complementary information (Bornette et al. 1998a; Bornette and Arens 2002; Paran et al. 2005). The hydraulic method precisely quantifies the flow and direction of the exchange, whereas the macrophyte and groundwater invertebrate indices may reveal the relative importance of groundwater inputs in comparison to surface water flows and can help to distinguish the origin of groundwater.

### Method limitations and accuracy

The accuracy of the results provided by the method discussed herein is different for each metric. For hydrogeology, we find the traditional sources of error, such as the depth of the aquifer, discontinuities in the alluvial aquifer and measurement errors related to groundwater level and hydraulic conductivity. For hydraulics, the depth of the channels has been estimated to be up to 5 m based on the quality of the data provided by the National Company of Rhône. The possible clogging of the banks has been estimated from the results given by the models when they were available.

For invertebrates, the limitations of the method lie in: i) the time required for sampling, ii) the identification of species, which is also time consuming and requires a large panel of specialists; and finally, iii) the lack of knowledge about the ecological preferences of many groundwater species. For the Brégnier-Cordon sector and for most of the northern part of Europe and North America, another limit related to the use of groundwater fauna is their low diversity in biogeographic zones that were frozen during the last glaciations (Ferreira et al. 2007). Most populations of groundwater species disappeared under quaternary glaciers and recolonized these areas during the last 10 ky (Ferreira et al. 2007). This low species richness certainly reduces the efficiency of the groundwater invertebrate index. Future research must test this index in areas located outside of the quaternary glacial limit.

Aquatic vegetation surveys are not time consuming in comparison to analyses of aquatic invertebrates, as most species can be identified in the field. Furthermore, the ecological requirements of plants are usually rather narrow because of their sessile character, improving their indicative value. However, there are some limitations related to i) the paucity of wetlands in the floodplain and ii) the existence of poorly informed species, either because they are rare and not documented, or because they exhibit a broad ecological niche. Finally, it is still necessary to test whether these ecological profiles are usable for other river systems.

At this stage of our work, we know that our method works but remains perfectible. To improve the method and its accuracy, our future works will focus on synchronous data to reduce the risk of errors in the interpretation of results and to make the statistical analysis efficient. Furthermore, we have to better consider the scale of interpretation: the spatial resolution of the estimated infiltration and the macrophyte index which is about 1-km versus 100 m for the stygobite richness and abundance.

## Conclusions

The numerical modeling of groundwater/surface water interactions is not always efficient for large-scale river basins. In very well-instrumented areas, the exchanges between groundwater and surface water can be calculated from a spatial analysis of piezometric gradients, and they can be validated by the results given by models that have been implemented on a local scale. Biological markers that are or are not influenced by phreatic conditions and discharge fluctuations can also confirm these surface water/groundwater interactions. This novel approach provides accurate maps of surface water/groundwater exchanges and demonstrates that hydraulics and biological metrics provide complementary information, despite the absence of significant correlation between hydrology and groundwater fauna, both in terms of spatial scale and the quality of information obtained. The results of this method will provide relevant hydrological and biological information on the complex interactions between groundwater and surface waters on the scale of the entire course of large rivers for decisions makers.

In terms of water resource management, the correspondence between hydraulics and biological indicators can help to determine sensitive areas where groundwater should be preserved. This is the case for the pumping well located in the southern part of the study area close to the Guiers River on the left bank of the floodplain. The maximum groundwater flow has been estimated to be 10.774 m^3^/day over a 1-km distance on this left bank. Considering that the influence radius of the well is equal to 300 m, the extraction rate of the well (1.355 m^3^/day) for supplying drinking water cannot be increased more than twice. In the case of groundwater overextraction, the exchange will be reversed, and the flow of the river can reach the pumping well.

Future research will be required to validate the spatial correlation found between the metrics in another case study to take into account contrasting situations regarding latitude, climate, geological context, the biogeography of organisms and anthropogenic conditions. Similarly, transient conditions remain to be studied because surface water/groundwater interactions can be modified during the fall low-flow period. Another field of research should be developed to predict the modification of these hydraulic exchanges in the framework of climate changes. Indeed, the thermal modifications that are induced by global warming could have harsh repercussions on the water demand of human populations. For example, nuclear power plants would require increasing amounts of stenothermal groundwater, and the groundwater demand for agriculture during summer periods would significantly increase.

## Electronic supplementary material

Additional file 1: **Calculation of the trophic level of a wetland.** The relevé data (left table) is multiplied by the affinity of the species for each trophic class (center table) to obtain the right table, wherein the highest value (4.46) indicates the best estimation of the trophic level of the site. (ZIP 509 KB)
